# Current and Emerging Role of Monoclonal Antibody-Based First-Line Treatment in Advanced Gastro-Esophageal and Gastric Cancer

**DOI:** 10.3390/curroncol30100672

**Published:** 2023-10-20

**Authors:** Audrey Désilets, Reem Elkhoury, Ahmad Gebai, Mustapha Tehfe

**Affiliations:** 1 Department of Medicine, Université de Montreal, Montreal, QC H3T 1J4, Canada; 2 Hematology-Oncology, Oncology Center-Centre Hospitalier de l’Universite de Montreal, Montreal, QC H2X 0C1, Canada

**Keywords:** advanced gastric cancer, monoclonal antibodies, targeted therapy, immune-checkpoint inhibitors, zolbetuximab, Claudin 18.2

## Abstract

Gastric cancer is the fifth most common malignancy worldwide and one of the main causes of cancer-related death. While surgical treatment is the only curative option for early disease, many have inoperable or advanced disease at diagnosis. Treatment in this case would be a combination of chemotherapy and immunotherapy. Gastro-esophageal (GEJ) and gastric cancer (GC) genetic profiling with current molecular diagnostic techniques has significantly changed the therapeutic landscape in advanced cancers. The identification of key players in GEJ and GC survival and proliferation, such as human epidermal growth factor 2 (HER2), vascular endothelial growth factor (VEGF), and programmed cell death protein 1 (PD-1)/programmed cell death ligand-1 (PD-L1), has allowed for the individualization of advanced cancer treatment and significant improvement in overall survival and progression-free survival of patients. This review comprehensively examines the current and emerging role of monoclonal antibody-based first-line treatments in advanced GEJ and GC. We explore the impact of monoclonal antibodies targeting HER2, VEGF, PD-1/PD-L1, and Claudin 18.2 (CLDN18.2) on the first-line treatment landscape by talking about key clinical trials. This review emphasizes the importance of biomarker testing for optimal treatment selection and provides practical recommendations based on ASCO guidelines.

## 1. Introduction

Gastric cancer, including adenocarcinoma of the stomach and gastro-esophageal junction (GEJ), is one of the most prevalent malignancies [1]. It also represents the third leading cause of cancer-related death worldwide [1,2,3]. Despite recent improvements in treatment options, the outcome of patients with advanced gastric cancer remains poor. Up to a third of those diagnosed with gastric cancer present with an advanced and unresectable disease [2]. With a median survival of 10–12 months, fewer than 5% of patients are still alive five years after their diagnosis [3]. 

Gastric cancer can be categorized according to the Lauren and WHO classifications, both of which rely solely on histopathologic findings [4]. In 2014, The Cancer Genome Atlas (TCGA) proposed a molecular classification of gastric cancer and distinguished four subtypes of gastric tumors: EBV positive tumors, microsatellite unstable tumors, genomically stable tumors, and tumors with chromosomal instability [5]. This classification allowed for a better understanding of the pathogenesis of the different subtypes of gastric cancer and helped uncover potential novel biomarkers. These biomarkers are of the uttermost importance in today’s era of precision medicine in oncology, as they provide clues to new targeted therapeutic agents. As an example, a loss of expression of mismatch-repair (MMR) genes results in an accumulation of mutations in microsatellites, which are short repeats of nucleotides distributed throughout the entire genome [6,7]. Tumors in which a loss of expression of two or more MMR genes is identified, either by polymerase chain reaction (PCR) or immunohistochemistry (IHC), are said to have high microsatellite instability (MSI-high/dMMR) [5,6,7,8]. As will be discussed later, an MSI-high status correlates with response to immunotherapy and confers a better prognosis; as such, it is critical that such testing be carried out thoroughly and accurately [9].

This review will focus on the role of monoclonal antibodies in the treatment of advanced adenocarcinomas of the stomach and gastro-esophageal junction in the first-line setting. We included in our literature review studies that were practice-changing, beginning with the ToGA trial, published in 2010, which was the first study to investigate the role of a monoclonal antibody in advanced gastric cancer, and ending with a discussion on emerging treatment options with anti-CLDN18.2 targeted therapy. We only considered studies on first-line treatment for patients with advanced adenocarcinomas of the stomach and gastro-esophageal junction. 

## 2. Anti-HER2 Receptor

HER2, a member of the human epidermal growth factor receptor family, is an important biomarker involved in the carcinogenesis of many tumors, including gastric cancer [10,11,12]. HER2 receptors are present on the surface of non-cancerous cells and are activated when they bind to another receptor from the HER family via a process known as protein dimerization [13,14]. The dimerization of HER receptors results in the activation of many signaling pathways involved in cell growth and survival. With that in mind, it is easier to understand how HER2 overexpression can promote carcinogenesis: being overexpressed, receptors come together more frequently, dysregulating intracellular signaling cascades and leading to aberrant cellular growth and proliferation [14]. Reported rates of HER2 overexpression in patients with gastric cancer vary from 10 to 30%, with a higher rate of HER2 positivity in GEJ or stomach cardia tumors [10,11,12,15]. HER2 overexpression is also more prevalent in the intestinal type by Lauren’s classification and in well- to moderately differentiated gastric cancers. Previous studies showed that HER2 overexpression was an independent prognostic marker, correlating with tumor size, serosal invasion, and lymph-node positive disease as well as a higher risk of recurrence and a reduced overall survival [16,17,18].

HER2 status can be either determined by immunohistochemistry (IHC) to assess protein overexpression, or by fluorescence in situ hybridization (FISH) to test for HER2 gene amplification [10,11,12,19]. Accurately determining the HER2 status of GEJ or GC is crucial as HER2-positive patients can benefit from the addition of the monoclonal antibody trastuzumab to their first-line systemic chemotherapy regimen (Table 1). This combination stems from the pivotal ToGA (Trastuzumab for Gastric Cancer) trial and is now considered a standard of care for this population of patients [15]. In the ToGA trial, the addition of trastuzumab to systemic chemotherapy, combining cisplatin and 5-FU, showed a significant improvement in overall survival (OS) from 11.1 to 13.8 months in HER2-positive patients with inoperable or advanced disease. Trastuzumab was subsequently the first molecular targeted agent to be approved in gastric cancer and significantly influenced the field of oncology, paving the way for the development of other targeted therapies for GEJ and GC.

Since then, other anti-HER2 agents have also been studied (Table 1). In the JACOB trial, the addition of pertuzumab to trastuzumab and chemotherapy did not provide a survival benefit [20]. Similarly, in the LOGiC trial, the addition of lapatinib, a tyrosine kinase inhibitor, to standard chemotherapy did not significantly improve OS [21]. Antibody-drug conjugates, such as trastuzumab-deruxtecan, have also been developed and showed improvements in response and OS in previously treated patients who remain HER2-positive. DESTINY-Gastric02, a single-arm, phase 2 study, recruited 89 patients with advanced, unresectable or metastatic HER2-positive gastric or GEJ cancer after progression on or after a first-line trastuzumab-containing regimen [24]. After a median follow-up of 10.2 months, an objective response was seen in 42% of patients, a finding that was clinically meaningful and supportive of trastuzumab-deruxtecan use in the second-line setting. However, trastuzumab-deruxtecan has yet to be tested in the first-line setting.

In summary, the accurate detection of HER2 overexpression is of uttermost importance in advanced, unresectable, and metastatic gastric cancer as it allows patients to benefit from the anti-HER2 monoclonal antibody trastuzumab in the first-line setting, in combination with a standard platinum-containing chemotherapy regimen. For now, no other anti-HER2 molecules are approved in the first-line setting.

## 3. Anti-VEGF

Another signal protein worthy of mention when examining the use of monoclonal antibodies in gastric cancer is vascular endothelial growth factor (VEGF). VEGF is a protein that cells produce and release in the interstitial fluid surrounding them to induce vessel growth [25]. Tumor cells are no exception to the rule and need nutrients to remain viable. Thus, angiogenesis is critical to carcinogenesis, and tumor cells also secrete VEGF to maintain a blood supply. It is in that context that anti-vascular endothelial growth factor therapies have garnered significant attention in the field of oncology (Table 1). Bevacizumab, notably, was the first monoclonal antibody targeting VEGF to make its appearance on the market [26]. More precisely, it is a humanized immunoglobulin G that recognizes and binds VEGF. VEGF being neutralized, it cannot attach to its receptor nor activate the cascade that would ultimately lead to vascular growth and tumor survival. After proving bevacizumab’s safety and efficacy in various tumor types, including colorectal cancer, it was studied in gastric cancer. The AVAGAST study, a prospective, randomized, double-blind, placebo-controlled phase 3 trial, was initiated to assess the impact of bevacizumab on treatment-naïve patients with locally advanced unresectable or metastatic gastric cancer [22]. While this study failed to meet its primary endpoint of a 2.8-month improvement in OS, it did demonstrate favorable outcomes in PFS and ORR. Moreover, although effective in first-line treatment of hepatocellular carcinoma, the combination of anti-VEGF and anti-PD-1/PD-L1 agents (which will be discussed in the next section of this review) has yet to be studied in first-line treatment of advanced GEJ and GC [27].

Another approved option is ramucirumab, an anti-VEGF receptor 2 (anti-VEGFR2) agent, which can be used as monotherapy or in combination with paclitaxel for patients with locally advanced unresectable or metastatic gastric cancer who have failed first-line treatment [28]. However, RAINFALL, a randomized phase 3 trial evaluating the addition of ramucirumab to cisplatin plus fluoropyrimidine chemotherapy, showed negative results [23]. Although the administration of ramucirumab led to a statistically significant improvement in investigator-assessed PFS, the clinical significance of this improvement was minimal, reflected in only 0.3-month difference in median PFS. Furthermore, the introduction of ramucirumab into first-line chemotherapy did not result in improved overall survival across any patient subgroup. Regrettably, the trial was unable to pinpoint a validated biomarker that could prospectively identify patients who would benefit most from ramucirumab treatment. Notably, the combination of ramucirumab with cisplatin-fluoropyrimidine was generally well tolerated, with manageable side effects. However, it is important to mention that there was a higher incidence of gastrointestinal perforations in the ramucirumab-treated group, although the majority of affected patients did recover.

Targeting angiogenic ligands and/or receptors combined with chemotherapy failed to improve survival outcomes in patients with advanced/metastatic GEJ or gastric cancer adenocarcinoma, and, therefore, it is not considered part of the first-line treatment armamentarium.

Table 1 summarizes the key phase 3 clinical trials just discussed regarding anti-HER2, anti-VEGF, and anti-VEGFR2 agents.

## 4. Anti-PD-1/PD-L1

Programmed death-1 (PD-1) is an inhibitory checkpoint receptor protein mainly expressed on the T cell surface [29]. When bound to programmed death-ligand 1 (PD-L1), it induces the downregulation of immune response, ultimately promoting immune evasion and tumor growth. The introduction of monoclonal antibodies targeting these checkpoint proteins, called immune-checkpoint inhibitors (ICI), has changed the treatment landscape of several cancers [30]. In gastric cancer, the inhibition of the PD-1/PD-L1 pathway with ICI including pembrolizumab and nivolumab has led to durable responses and improved survival in chemo-refractory patients [31].

Before proceeding further, we must emphasize that biomarkers, such as PD-L1 combined positive score (CPS) and tumor mutational burden (TMB), have been developed to help better predict responses to immunotherapy [32,33]. First, PD-L1 expression is determined by IHC staining and is reported using a combined positive score (CPS), which is defined as the proportion of all tumor cells that stain for PD-L1 on immunohistochemistry, multiplied by 100 [32]. Tumors are PD-L1-positive if the CPS is ≥1. On the other hand, TMB estimates the number of mutations in a cell’s DNA and is assessed on a biopsy specimen using next-generation sequencing [33]. However, authors disagree regarding the cut-off value that should be used to separate “TMB-high” from “TMB-low” tumors, a correlation between TMB-high tumors and responses to immunotherapy in gastric cancer has yet to be made, and the technique is not readily accessible [34]. Nonetheless, PD-L1 CPS remains the most important predictive biomarker of responses to immunotherapy. The estimated prevalence of PD-L1 expression in patients with gastroesophageal adenocarcinomas ranges between 40% and 57%, as reported, respectively, in KEYNOTE-012 and KEYNOTE-059, both early phase trials to evaluate the safety of pembrolizumab in patients with gastric cancer [35,36].

The efficacy of ICI in the first-line setting for patients with advanced GC has been the subject of several trials (Table 2). The discussion presented in the following paragraphs will summarize trials and clinical data supporting the use of immunotherapy in HER2-negative advanced gastric cancer.

In the randomized phase 3 trial CheckMate-649, the role of combined immunotherapy and cytotoxic chemotherapy as the initial therapeutic regimen for patients with advanced GC was investigated [37]. In this study, a total of 1581 patients with previously untreated advanced GC (70%), GEJ (18%), or esophageal (12%) adenocarcinoma were randomly assigned to nivolumab plus chemotherapy or chemotherapy alone. All patients had a HER2-negative status, and nivolumab was added independently of their PD-L1 status. At a minimum follow-up of 12.1 months, a significant 29% decrease in risk of death was observed in patients with a PD-L1 CPS of 5 or more when nivolumab was added to chemotherapy. Moreover, there was a significant improvement of 3.3 months in the median OS (mOS) between the two groups (14.4 vs. 11.1 months). A median progression-free survival (PFS) of 7.7 months was observed in the nivolumab group as opposed to 6.05 months in the placebo group. CheckMate-649 was thus the first study to show an improved survival in HER2-negative advanced GEJ/GC with the combination of ICI and chemotherapy in the first-line setting, irrespective of PD-L1 score. This allowed for the approval of nivolumab in 2021 in combination with fluoropyrimidine and platinum-containing chemotherapy, which currently still represents the standard of care for first-line treatment for this subset of patients.

Updated data from the 2023 Gastrointestinal Cancers Symposium show that at a minimum follow-up of 36.2 months, the addition of nivolumab to chemotherapy elicits OS and PFS benefits both in the population of patients with a PD-L1 CPS of ≥5 (representing 60% of the study population, *n* = 955) and in the all-randomized population [38]. Patients exposed to combination therapy had a median OS of 14.4 months versus 11.1 months with chemotherapy alone (HR 0.70; 95% CI 0.61–0.81). The 3-year survival rate was 21 vs. 10%, with the survival curve reaching a plateau after 36 months. PFS was also prolonged from 6.1 to 8.3 months (HR 0.79; 95% CI 0.60–0.81) in the combination arm. In the same way, clinically meaningful OS improvement was seen in the all-randomized population with a 3-year survival rate of 17 vs. 10%. The combination of nivolumab and chemotherapy also significantly prolonged OS (13.7 vs. 11.6 months; HR 0.79; 95% CI 0.71–0.88) and PFS (7.7 vs. 6.9 months; HR 0.79; 95% CI 0.71–0.89) vs. chemotherapy alone. 

The efficacy and safety of sintilimab, another anti-PD-1 monoclonal antibody, was evaluated in the Asian phase 3 ORIENT-16 trial in which 650 patients with advanced gastric adenocarcinoma were randomized to receive either sintilimab plus CAPOX or placebo plus CAPOX [39]. The addition of sintilimab to chemotherapy showed a superior OS benefit in patients with CPS ≥ 5 (mOS 18.4 vs. 12.9 months; HR 0.66; 95% CI 0.50–0.86) and among all patients (mOS 15.2 vs. 12.3 months; HR 0.76; 95% CI 0.62–0.93). These findings were consistent with the CHECKMATE-649 trial: regardless of PD-L1 expression, patients seem to benefit from combination therapy. However, we observe lower HRs when patients whose tumors express low CPS scores are added to the analyses. Similarly, tislelizumab, also an anti-PD-1 monoclonal antibody, met the primary endpoint of overall survival in the phase 3 study RATIONALE 305 [40]. In combination with chemotherapy, tislelizumab significantly improved OS when used in the frontline treatment of patients with PD-L1-positive (PD-L1 score ≥ 5% of tumor cells) locally advanced, unresectable, or metastatic gastric or GEJ adenocarcinoma (mOS 17.2 vs. 12.6 months; HR 0.74; 95% CI 0.59–0.94). Tislelizumab also improved PFS, duration of response, and objective response rate compared to chemotherapy plus placebo. The safety profile was consistent with what was previously observed.

ATTRACTION-4, a randomised, phase 3 trial, compared nivolumab combined with oxaliplatin-based chemotherapy to chemotherapy alone in untreated Asian patients (Japan, South Korea, and Taiwan) with HER2-negative, unresectable advanced or recurrent gastric or GEJ cancer [41]. Although there was a significant improvement in PFS (10.94 vs. 8.41 months; HR 0.70, *p* = 0.0005) at final analysis, the median survival in both groups was similar over 17 months. It is worth mentioning the following explanation for the discrepancy with CheckMate-649. First, although it is hard to compare PD-L1 tumor proportion score (TPS) to CPS, 85% of the ATTRACTION-4 patients had a TPS PD-L1 < 1%, while in ChekMate-649 more than 60% had a CPS > 5. Second, subsequent treatments after progression were seen in more than 70% of the participants in ATTRACTION-4 compared to 39% in the ChekMate-649 groups.

The role of pembrolizumab was also investigated in the phase 3 KEYNOTE-859 trial [42]. A total of 1579 previously untreated patients with advanced adenocarcinomas of the stomach and GEJ were randomized to receive either pembrolizumab or placebo, both given with a standard-of-care chemotherapy, at the choice of the investigator (86% of patients received a capecitabine plus oxaliplatin regimen, and the remainder fluorouracil plus cisplatin). At a median follow-up of 31 months, the median OS was 12.9 months with pembrolizumab compared with 11.5 months with chemotherapy alone, showing a benefit of the addition of pembrolizumab regardless of PD-L1 expression. The median PFS was 6.9 vs. 5.6 months. All subgroups of patients seemed to benefit from the addition of pembrolizumab, those with microsatellite-high tumors and a PD-L1 CPS ≥ 10% deriving the most important relative reduction in the risk of death (66% and 36%, respectively, vs. 22% in the overall population). Thus, the findings of KEYNOTE-859 support pembrolizumab as another treatment option for locally advanced, unresectable, or metastatic gastric and GEJ adenocarcinomas, and reinforces previous trials showing the benefit of adding ICI to chemotherapy. 

Here, it must be mentioned that pembrolizumab in gastric cancer was also studied in the KEYNOTE-062 trial which missed its primary OS endpoint [43]. In this study, a total of 763 patients with PD-L1-positive (CPS ≥ 1) advanced gastric or GEJ adenocarcinoma were randomly assigned between three groups: pembrolizumab monotherapy, pembrolizumab combined with chemotherapy, or chemotherapy alone. In the combined pembrolizumab and chemotherapy group, the mOS was of 12.5 months (95% CI, 10.8–13.9 months), compared with 11.1 months in the chemotherapy group (95% CI, 9.2–12.8 months), thus not meeting the criteria for superiority (HR 0.85; 95% CI 0.70–1.03). The analysis of median PFS similarly did not show superiority in the study (6.9 vs. 6.4 months; HR 0.84; 95% CI 0.70–1.00).

Even more surprisingly, the pembrolizumab plus chemotherapy combination did not show any benefit in OS or PFS in the CPS ≥ 10% population. Why is that the case? Perhaps the best explanation lies in the interactions between chemotherapy and immunotherapy [44]. KEYNOTE-062 was the only large clinical trial investigating the role of pembrolizumab in gastric cancer using cisplatin instead of oxaliplatin as a platinum-based agent. This raises the possibility that the use of cisplatin might result in some degree of resistance to immunotherapy and alter the outcomes in patients exposed to anti-PD-1 antibodies. In the PD-L1 CPS ≥ 10% subgroup, pembrolizumab in monotherapy provided a clinically significant gain in OS (mOS 17.4 vs. 11.1 months; HR 0.62; 95% CI 0.45–0.86) as well as a greater 24-month OS rate (28.3%) compared to the chemotherapy arm (21.2%). The survival benefit was even more pronounced among patients with MSI-high tumors: the median OS with pembrolizumab was not reached in MSI-high patients with PD-L1 CPS ≥ 1 vs. 8.5 months (95% CI 5.3–20.8) in the chemotherapy group. Similar findings were found in the PD-L1 CPS ≥ 10 population (mOS not reached vs. 13.6 months (95% CI 3.8–25.8)). Although it was known from previous studies, such as CheckMate-649, that the subgroup of patients with MSI-high tumors do better than the others, KEYNOTE-062 was the first study to show a survival benefit from pembrolizumab in monotherapy for MSI-high tumors in the first-line setting, independently of the PD-L1 CPS value. Whether these patients need a combination treatment with chemotherapy or not is still debated. 

Pembrolizumab was also investigated in the treatment of HER2-positive advanced gastric cancer. Preclinical studies demonstrated that combining trastuzumab and ICI reinforces immune cell reaction and particularly HER-2-specific T cell responses [45,46]. The PANTHERA trial, a multicenter phase 1b/2 trial, investigated the combination of trastuzumab, pembrolizumab, and chemotherapy in advanced HER2-postiive gastric cancer [47]. A total of 38 patients were included, all of whom were microsatellite-stable and EBV-negative; 55% of patients had a PD-L1 CPS ≥ 1. With a median PFS of 8.6 months and a median OS of 19.3 months, the results, which were presented at ASCO 2021, were promising, and led to the phase 3 trial KEYNOTE-811 [48]. In this phase 3 trial, 434 patients were randomized between trastuzumab/pembrolizumab and platinum-based chemotherapy vs. placebo/pembrolizumab and chemotherapy. Interim data were recently reported and showed a significantly improved objective response rate of 74.4% in the pembrolizumab group vs. 51.9% in the control group. The complete response rate was also higher among patients receiving pembrolizumab (11.3% vs. 3.1%). Based on this analysis, the FDA approved the dual PD-1 and HER2 blockade in HER2-positive gastric or GEJ previously untreated and advanced adenocarcinoma. The awaited survival outcomes will be presented soon at ESMO 2023.

The following table summarizes the key phase 3 clinical trials just discussed regarding anti-PD-1/PD-L1 agents.

**Table 2:** Key phase 3 clinical trials for anti-PD-1/PD-L1 and summary of their results for first-line immunotherapy in advanced GC/GEJ cancers.

Trial	Agent	Experimental Arm	Control Arm	Primary Endpoints	Results (Experimental vs. Control)	Reference in the Text
CheckMate-649	Nivolumab	Nivolumab + chemotherapy (XELOX or FOLFOX)	XELOX or FOLFOX	OS and PFS in patients with CPS ≥ 5	OS: 14.4 vs. 11.1 months (HR 0.71; 98.4% CI 0.59–0.86) PFS: 7.7 vs. 6.05 months (HR 0.68; 98% CI 0.56–0.81)	[37]
ORIENT-16	Sintilimab	Sintilimab + chemotherapy (CAPOX)	CAPOX	OS in patients with CPS ≥ 5 and OS in all patients	OS in patients with CPS ≥ 5: 18.4 vs. 12.9 months (HR 0.660; 95% CI 0.505–0.864) OS in all patients: 15.2 vs. 12.3 months (HR 0.766; 95% CI 0.626–0.936)	[39]
RATIONALE 305	Tislelizumab	Tislelizumab + chemotherapy (CAPOX or cisplatin + 5-FU)	CAPOX or cisplatin + 5-FU	OS	OS: 17.2 vs. 12.6 months (HR 0.74; 95% CI 0.59–0.94)	[40]
ATTRACTION-4	Nivolumab	Nivolumab + chemotherapy (SOX or CAPOX)	SOX or CAPOX	PFS and OS	PFS: 10.45 vs. 8.34 months (HR 0.68; 98.51% CI 0.51–0.90) OS: 17.45 vs. 17.15 months (HR 0.90; 95% CI 15.67–20.83)	[41]
KEYNOTE-859	Pembrolizumab	Pembrolizumab + chemotherapy (cisplatin + 5-FU or CAPOX)	Cisplatin + 5-FU or CAPOX	OS	OS: 12.9 vs. 11.5 months (HR 0.78; 95% CI 0.70–0.87)	[42]
KEYNOTE-062	Pembrolizumab	Pembrolizumab or pembrolizumab + chemotherapy (cisplatin + 5-FU or cisplatin + capecitabine)	Cisplatin + 5-FU or cisplatin + capecitabine	OS and PFS in patients with CPS ≥ 1	Pembrolizumab vs. chemotherapy OS: 10.6 vs. 11.1 months (HR 0.91; 99.2% CI 0.69–1.18) PFS: 2.0 vs. 6.4 months (HR 1.66; 95% CI 1.37–2.01) Pembrolizumab + chemotherapy vs. chemotherapy: OS: 12.5 vs. 11.1 months (HR 0.85; 95% CI 0.70–1.03) PFS: 6.9 vs. 6.4 months (HR 0.84; 95% CI 0.70–1.02)	[43]
KEYNOTE-811	Trastuzumab + pembrolizumab	Trastuzumab + pembrolizumab + chemotherapy (cisplatin + 5-FU or CAPOX)	Cisplatin + 5-FU or CAPOX	OS and PFS	Interim results ORR: 74.4% vs. 51.9%	[48]

## 5. Anti-Claudin 18.2

Claudins (CLDN) form a family of 27 transmembrane proteins that play crucial roles in intercellular tight junctions [49,50,51]. The overexpression of CLDNs has been reported to increase aberrant localization and function in various cancer types, promoting metastasis and progression [52,53]. CLDN18.2 is a tight junction protein that is normally exclusively expressed in gastric mucosa cells [54]. In gastric and GEJ adenocarcinomas, CLDN18.2 expression is commonly retained. A loss of cell polarity during malignant transformation may expose CLDN18.2 on the cell surface, rendering it more accessible to antibodies, and has thus recently become a promising emerging therapeutic target [55,56,57]. The CLDN18.2 status and degree of expression can be determined by IHC. Reported rates of moderate to strong CLDN18.2 overexpression in gastric and GEJ cancers vary between 30 and 52% [58,59].

Zolbetuximab is a first-in-class immunoglobulin monoclonal antibody that targets CLDN18.2 and mediates immune lysis via complement-dependent toxicity and antibody-dependent cellular toxicity [56,60,61]. The recent phase 3 trial SPOTLIGHT investigated the role of first-line zolbetuximab combined with cytotoxic chemotherapy in advanced gastric or GEJ cancers [62]. All 565 patients with a CLDN18.2-positive (≥75% of tumor cells with moderate-to-strong CLDN18.2 expression), HER2-negative, previously untreated locally advanced unresectable or metastatic gastric or GEJ adenocarcinoma were randomly assigned to mFOLFOX6 combined with zolbetuximab or placebo. The combination of zolbetuximab and mFOLFOX6 resulted in a clinically significant improvement in median PFS of 10.61 vs. 8.67 months (HR 0.751; 95% CI 0.589–0.941; *p* = 0.0066) in the placebo group. The median OS in the Zolbetuximab arm was 18.23 months compared to 15.54 months in the placebo group (HR 0.750; 95% CI 0.601–0.936; *p* = 0.0053). Both the objective response rate (60.7 vs. 62.1%) and the duration of response (8.51 vs. 8.11 months) were similar in both groups. Grade 3 or worse adverse events led to the discontinuation of the treatment in 14% of patients in the zolbetuximab group and in 6% of patients in the placebo group.

In March 2023, the ASCO Plenary Series featured the primary results from another phase 3 trial, the GLOW study [63], which sought to investigate the role of first-line addition of zolbetuximab to CAPOX chemotherapy on PFS when compared to chemotherapy alone. A total of 507 patients with a CLDN18.2-positive but HER2-negative status with advanced unresectable or metastatic gastric or GEJ adenocarcinoma were blindly assigned to either group. The combination of zolbetuximab with chemotherapy resulted in a notable increase in PFS, validating the findings of the aforementioned SPOTLIGHT study. Indeed, an improvement in PFS from 6.80 months in the placebo group to 8.21 months in the zolbetuximab group was observed (HR 0.687; 95% CI 0.544–0.866; *p* = 0.0007). Similarly, OS was also significantly longer in the combination group (14.39 vs. 12.16 months; HR 0.771; 95% CI 0.615–0.965; *p* = 0.0118). ORR was noted in 53.8% of the zolbetuximab arm compared to 48.8% in the placebo arm. Treatment-related adverse events led to zolbetuximab discontinuation in 7.1% of patients, compared with 4.4% in the placebo arm.

Taken together, these two phase 3 studies show a promising clinically significant benefit for use of the first anti-CLDN18.2, zolbetuximab, for treatment of locally advanced unresectable or metastatic gastric or GEJ cancers with a CLDN18.2-positive, HER2-negative molecular profile. 

Table 3 below summarizes the phase 3 trials just discussed regarding anti-CLDN18.2.

## 6. Conclusions

In the first-line setting, monoclonal antibodies have become a prevalent treatment for advanced adenocarcinomas of the stomach and gastro-esophageal junction. It is of utmost importance that all newly diagnosed patients are tested for HER2 status, MSI status, and PD-L1 expression using CPS scoring, as these are the biomarkers that currently guide the selection of the optimal first-line treatment regimen. This review pinpoints some of the most important trials that have taken place in the last few years and underlines key recommendations extracted from the ASCO guidelines [64]. Regarding PD-L1 expression, it must be pointed out that different trials used different PD-L1 cut-offs for their analyses. Therefore, the optimal PD-L1 cut-offs are not known. However, most international cancer societies’ guidelines use the cut-off value of CPS 5. Thus, in previously untreated, unresectable or metastatic gastric or GEJ adenocarcinomas:

(1) HER2-positive patients should be offered trastuzumab with cytotoxic chemotherapy (fluoropyrimidine and oxaliplatin-based regimen); adding pembrolizumab is an option but not yet widely available;
(2) HER2-negative patients with a PD-L1 CPS ≥ 5% should be offered nivolumab, or pembrolizumab for PD-L1 CPS ≥ 10%, in combination with cytotoxic chemotherapy (fluoropyrimidine and platinum-based regimen);
(3) HER2-negative patients with a PD-L1 CPS 1–5 should be offered cytotoxic chemotherapy (fluoropyrimidine and platinum-based regimen), as well as considered for the addition of nivolumab;

HER2-negative patients with a PD-L1 CPS 0 should be offered cytotoxic chemotherapy (fluoropyrimidine and platinum-based regimen), without the addition of immunotherapy.

Furthermore, as two recent phase 3 trials showed a promising benefit for the use of the first anti-CLDN18.2, zolbetuximab, patients should also be assessed at diagnosis for CLDN18.2 positivity. Thus, only few patients will be treated with chemotherapy only.

Currently, and despite all these therapeutic options, we are far from curing GEJ and GC patients. The next challenge for basic science researchers and clinicians is to refine molecular and clinical biomarkers for a better treatment selection. Inviting patients to participate in clinical trials exploring new combinations with anti-FGFR2b (fibroblast growth factor receptor 2b), anti-TIGIT (T cell immunoreceptor with immunoglobulin and ITIM domain), or anti-LAG3 (lymphocyte activation gene 3), among many other cancer targets, is to be encouraged.

## Figures and Tables

**Table 1:** Key phase 3 clinical trials for anti-HER2, anti-VEGF, and anti-VEGFR-2 combined with chemotherapy in first-line treatment in advanced GC/GEJ cancers.

Target	Trial	Agent	Experimental Arm	Control Arm	Primary Endpoints	Results (Experimental vs. Control)	Reference in the Text
HER2	ToGA	Trastuzumab	Trastuzumab + chemotherapy (cisplatin + 5-FU or cisplatin + capecitabine)	Cisplatin/5-FU or cisplatin/capecitabine	OS	OS: 13.8 vs. 11.1 months (HR 0.74; 95% CI 0.60–0.91)	[15]
	JACOB	Pertuzumab + trastuzumab	Pertuzumab + Trastuzumab + chemotherapy (cisplatin or capecitabine or 5-FU)	Trastuzumab + chemotherapy (cisplatin or capecitabine or 5-FU)	OS	OS: 17.5 vs. 14.2 months (HR 0.84; 95% CI 0.71–1.00)	[20]
	LOGiC	Lapatinib	Lapatinib + chemotherapy (capecitabine + oxaliplatin)	Capecitabine + oxaliplatin	OS	OS: 12.2 vs. 10.5 months (HR 0.91; 95% CI 0.73–1.12)	[21]
VEGF	AVAGAST	Bevacizumab	Bevacizumab + chemotherapy (cisplatin + capecitabine or cisplatin + 5-FU)	Cisplatin + capecitabine or cisplatin + 5-FU	OS	OS: 12.1 vs. 10.1 months (HR 0.87; 95% CI 0.73–1.03)	[22]
VEGFR-2	RAINFALL	Ramucirumab	Ramucirumab + chemotherapy (cisplatin + capecitabine or cisplatin + 5-FU)	Cisplatin + capecitabine or cisplatin + 5-FU	PFS	PFS: 6.7 vs. 5.4 months (HR 0.753; 85% CI 0.607–0.935)	[23]

**Table 3:** Key phase 3 clinical trials for anti-CLDN18.2 combined to chemotherapy in first-line treatment in advanced GC/GEJ cancers.

Trial	Agent	Experimental Arm	Control Arm	Primary Endpoint	Results (Experimental vs. Control)	Reference in the Text
SPOTLIGHT	Zolbetuximab	Zolbetuximab + chemotherapy (mFOLFOX6)	mFOLFOX6	PFS	PFS: 10.61 vs. 8.67 months (HR 0.751; 95% CI 0.589–0.941)	[62]
GLOW	Zolbetuximab	Zolbetuximab + chemotherapy (CAPOX)	CAPOX	PFS	PFS: 8.21 vs. 6.80 months (HR 0.687; 95% CI 0.544–0.866)	[63]

## References

[1] Smyth E.C., Nilsson M., Grabsch H.I., van Grieken N.C., Lordick F. (2020). Gastric cancer. Lancet.

[2] Jim M.A., Pinheiro P.S., Carreira H., Espey D.K., Wiggins C.L., Weir H.K. (2020). Stomach cancer survival in the United States by race and stage (2001–2009): Findings from the CONCORD-2 study. Cancer.

[3] Thrift A.P., El-Serag H.B. (2020). Burden of Gastric Cancer. Clin. Gastroenterol. Hepatol..

[4] Luebke T., Baldus S.E., Grass G., Bollschweiler E., Thiele J., Dienes H.P., Hoelscher A.H., Moenig S.P. (2005). Histological grading in gastric cancer by Ming classification: Correlation with histopathological subtypes, metastasis, and prognosis. World J. Surg..

[5] The Cancer Genome Atlas Research Network (2014). Comprehensive molecular characterization of gastric adenocarcinoma. Nature.

[6] Puliga E., Corso S., Pietrantonio F., Giordano S. (2021). Microsatellite instability in Gastric Cancer: Between lights and shadows. Cancer Treat. Rev..

[7] Li K., Luo H., Huang L., Luo H., Zhu X. (2020). Microsatellite instability: A review of what the oncologist should know. Cancer Cell Int..

[8] Cristescu R., Lee J., Nebozhyn M., Kim K.M., Ting J.C., Wong S.S., Liu J., Yue Y.G., Wang J., Yu K. (2015). Molecular analysis of gastric cancer identifies subtypes associated with distinct clinical outcomes. Nat. Med..

[9] Pietrantonio F., Miceli R., Raimondi A., Kim Y.W., Kang W.K., Langley R.E., Choi Y.Y., Kim K.M., Nankivell M.G., Morano F. (2019). Individual patient data meta-analysis of the value of microsatellite instability as a biomarker in gastric cancer. J. Clin. Oncol..

[10] Gravalos C., Jimeno A. (2008). HER2 in gastric cancer: A new prognostic factor and a novel therapeutic target. Ann. Oncol. Off. J. Eur. Soc. Med. Oncol..

[11] Cho E.Y., Park K., Do I., Cho J., Kim J., Lee J., Kim S., Kim K.M., Sohn T.S., Kang W.K. (2013). Heterogeneity of ERBB2 in gastric carcinomas: A study of tissue microarray and matched primary and metastatic carcinomas. Mod. Pathol..

[12] Choi S., Chu J., Kim B., Ha S.Y., Kim S.T., Lee J., Kang W.K., Han H., Sohn I., Kim K.M. (2019). Tumor Heterogeneity Index to Detect Human Epidermal Growth Factor Receptor 2 Amplification by Next-Generation Sequencing: A Direct Comparison Study with Immunohistochemistry. J. Mol. Diagn..

[13] Iqbal N., Iqbal N. (2014). Human Epidermal Growth Factor Receptor 2 (HER2) in Cancers: Overexpression and Therapeutic Implications. Mol. Biol. Int..

[14] Yan M., Schwaederle M., Arguello D., Millis S.Z., Gatalica Z., Kurzrock R. (2015). HER2 expression status in diverse cancers: Review of results from 37,992 patients. Cancer Metastasis Rev..

[15] Bang Y.J., van Cutsem E., Feyereislova A., Chung H.C., Shen L., Sawaki A., Lordick F., Ohtsu A., Omuro Y., Satoh T. (2010). Trastuzumab in combination with chemotherapy versus chemotherapy alone for treatment of HER2-positive advanced gastric or gastro-oesophageal junction cancer (ToGA): A phase 3, open-label, randomised controlled trial. Lancet.

[16] Jørgensen J.T., Hersom M. (2012). HER2 as a Prognostic Marker in Gastric Cancer—A Systematic Analysis of Data from the Literature. J. Cancer.

[17] Allgayer H., Babic R., Gruetzner K.U., Tarabichi A., Schildberg F.W., Heiss M.M. (2000). c-erbB-2 is of independent prognostic relevance in gastric cancer and is associated with the expression of tumor-associated protease systems. J. Clin. Oncol. Off. J. Am. Soc. Clin. Oncol..

[18] Tanner M., Hollmén M., Junttila T.T., Kapanen A.I., Tommola S., Soini Y., Helin H., Salo J., Joensuu H., Sihvo E. (2005). Amplification of HER-2 in gastric carcinoma: Association with Topoisomerase IIα gene amplification, intestinal type, poor prognosis and sensitivity to trastuzumab. Ann. Oncol..

[19] Choi S., Park S., Kim H., Kang S.Y., Ahn S., Kim K.M. (2022). Gastric Cancer: Mechanisms, Biomarkers, and Therapeutic Approaches. Biomedicines.

[20] Tabernero J., Hoff P.M., Shen L., Ohtsu A., Shah M.A., Siddiqui A., Heeson S., Kiermaier A., Macharia H., Restuccia E. (2023). Pertuzumab, trastuzumab, and chemotherapy in HER2-positive gastric/gastroesophageal junction cancer: End-of-study analysis of the JACOB phase III randomized clinical trial. Gastric Cancer.

[21] Hecht J.R., Bang Y.J., Qin S.K., Chung H.C., Xu J.M., Park J.O., Jeziorski K., Shparyk Y., Hoff P.M., Sobrero A. (2016). Lapatinib in combination with capecitabine plus oxaliplatin in human epidermal growth factor receptor 2-positive advanced or metastatic gastric, esophageal, or gastroesophageal adenocarcinoma: TRIO-013/LOGiC—A randomized phase III trial. J. Clin. Oncol..

[22] Ohtsu A., Shah M.A., van Cutsem E., Rha S.Y., Sawaki A., Park S.R., Lim H.Y., Yamada Y., Wu J., Langer B. (2011). Bevacizumab in combination with chemotherapy as first-line therapy in advanced gastric cancer: A randomized, double-blind, placebo-controlled phase III study. J. Clin. Oncol..

[23] Fuchs C.S., Shitara K., di Bartolomeo M., Lonardi S., Al-Batran S.E., van Cutsem E., Ilson D.H., Alsina M., Chau I., Lacy J. (2019). Ramucirumab with cisplatin and fluoropyrimidine as first-line therapy in patients with metastatic gastric or junctional adenocarcinoma (RAINFALL): A double-blind, randomised, placebo-controlled, phase 3 trial. Lancet Oncol..

[24] van Cutsem E., di Bartolomeo M., Smyth E., Chau I., Park H., Siena S., Lonardi S., Wainberg Z.A., Ajani J., Chao J. (2023). Trastuzumab deruxtecan in patients in the USA and Europe with HER2-positive advanced gastric or gastroesophageal junction cancer with disease progression on or after a trastuzumab-containing regimen (DESTINY-Gastric02): Primary and updated analyses from a single-arm, phase 2 study. Lancet Oncol..

[25] Shibuya M. (2011). Vascular Endothelial Growth Factor (VEGF) and Its Receptor (VEGFR) Signaling in Angiogenesis: A Crucial Target for Anti- and Pro-Angiogenic Therapies. Genes Cancer.

[26] Ferrara N., Hillan K.J., Novotny W. (2005). Bevacizumab (Avastin), a humanized anti-VEGF monoclonal antibody for cancer therapy. Biochem. Biophys. Res. Commun..

[27] Finn R.S., Qin S., Ikeda M., Galle P.R., Ducreux M., Kim T.-Y., Kudo M., Breder V., Merle P., Kaseb A.O. (2020). Atezolizumab plus Bevacizumab in Unresectable Hepatocellular Carcinoma. N. Engl. J. Med..

[28] di Bartolomeo M., Niger M., Tirino G., Petrillo A., Berenato R., Laterza M.M., Pietrantonio F., Morano F., Antista M., Lonardi S. (2018). Ramucirumab as Second-Line Therapy in Metastatic Gastric Cancer: Real-World Data from the RAMoss Study. Target. Oncol..

[29] Han Y., Liu D., Li L. (2020). PD-1/PD-L1 pathway: Current researches in cancer. Am. J. Cancer Res..

[30] Shiravand Y., Khodadadi F., Kashani SM A., Hosseini-Fard S.R., Hosseini S., Sadeghirad H., Ladwa R., O’byrne K., Kulasinghe A. (2022). Immune Checkpoint Inhibitors in Cancer Therapy. Curr. Oncol..

[31] Chan W.L., Lam K.O., So T.H., Lee V.H.F., Kwong L.W.D. (2019). Third-line systemic treatment in advanced/metastatic gastric cancer: A comprehensive review. Ther. Adv. Med. Oncol..

[32] Akhtar M., Rashid S., Al-Bozom I.A. (2021). PD−L1 immunostaining: What pathologists need to know. Diagn. Pathol..

[33] Fusco M.J., West H.J., Walko C.M. (2021). Tumor Mutation Burden and Cancer Treatment. JAMA Oncol..

[34] Folprecht G. (2019). Tumor mutational burden as a new biomarker for PD-1 antibody treatment in gastric cancer. Cancer Commun..

[35] Muro K., Chung H.C., Shankaran V., Geva R., Catenacci D., Gupta S., Eder J.P., Golan T., Le D.T., Burtness B. (2016). Pembrolizumab for patients with PD-L1-positive advanced gastric cancer (KEYNOTE-012): A multicentre, open-label, phase 1b trial. Lancet Oncol..

[36] Fuchs C.S., Doi T., Jang R.W., Muro K., Satoh T., Machado M., Sun W., Jalal S.I., Shah M.A., Metges J.P. (2018). Safety and Efficacy of Pembrolizumab Monotherapy in Patients with Previously Treated Advanced Gastric and Gastroesophageal Junction Cancer: Phase 2 Clinical KEYNOTE-059 Trial. JAMA Oncol..

[37] Janjigian Y.Y., Shitara K., Moehler M., Garrido M., Salman P., Shen L., Wyrwicz L., Yamaguchi K., Skoczylas T., Campos Bragagnoli A. (2021). First-line nivolumab plus chemotherapy versus chemotherapy alone for advanced gastric, gastro-oesophageal junction, and oesophageal adenocarcinoma (CheckMate 649): A randomised, open-label, phase 3 trial. Lancet.

[38] Janjigian Y.Y., Shitara K., Moehler M.H., Garrido M., Gallardo C., Shen L., Yamaguchi K., Wyrwicz L., Skoczylas T., Campos Bragagnoli A.S. (2023). Nivolumab (NIVO) plus chemotherapy (chemo) vs chemo as first-line (1L) treatment for advanced gastric cancer/gastroesophageal junction cancer/esophageal adenocarcinoma (GC/GEJC/EAC): 3-year follow-up from CheckMate 649. J. Clin. Oncol..

[39] Xu J., Jiang H., Pan Y., Gu K., Cang S., Han L., Shu Y., Li J., Zhao J., Pan H. (2021). LBA53 Sintilimab plus chemotherapy (chemo) versus chemo as first-line treatment for advanced gastric or gastroesophageal junction (G/GEJ) adenocarcinoma (ORIENT-16): First results of a randomized, double-blind, phase III study. Ann. Oncol..

[40] Moehler M.H., Kato K., Arkenau H.-T., Oh D.-Y., Tabernero J., Cruz-Correa M., Wang H., Xu H., Li J., Yang S. (2023). Rationale 305: Phase 3 study of tislelizumab plus chemotherapy vs placebo plus chemotherapy as first-line treatment (1L) of advanced gastric or gastroesophageal junction adenocarcinoma (GC/GEJC). J. Clin. Oncol..

[41] Kang Y.K., Chen L.T., Ryu M.H., Oh D.Y., Oh S.C., Chung H.C., Lee K.W., Omori T., Shitara K., Sakuramoto S. (2022). Nivolumab plus chemotherapy versus placebo plus chemotherapy in patients with HER2-negative, untreated, unresectable advanced or recurrent gastric or gastro-oesophageal junction cancer (ATTRACTION-4): A randomised, multicentre, double-blind, placebo-controlled, phase 3 trial. Lancet Oncol..

[42] Tabernero J., Bang Y.J., van Cutsem E., Fuchs C.S., Janjigian Y.Y., Bhagia P., Li K., Adelberg D., Qin S.K. (2021). KEYNOTE-859: A Phase III study of pembrolizumab plus chemotherapy in gastric/gastroesophageal junction adenocarcinoma. Future Oncol..

[43] Shitara K., van Cutsem E., Bang Y.J., Fuchs C., Wyrwicz L., Lee K.W., Kudaba I., Garrido M., Chung H.C., Lee J. (2020). Efficacy and Safety of Pembrolizumab or Pembrolizumab Plus Chemotherapy vs Chemotherapy Alone for Patients With First-line, Advanced Gastric Cancer: The KEYNOTE-062 Phase 3 Randomized Clinical Trial. JAMA Oncol..

[44] Emens L.A., Middleton G. (2015). The Interplay of Immunotherapy and Chemotherapy: Harnessing Potential Synergies. Cancer Immunol. Res..

[45] Park S.G., Jiang Z., Mortenson E.D., Deng L., Radkevich-Brown O., Yang X., Sattar H., Wang Y., Brown N.K., Greene M. (2010). The therapeutic effect of anti-HER2/neu antibody depends on both innate and adaptive immunity. Cancer Cell.

[46] Stagg J., Loi S., Divisekera U., Ngiow S.F., Duret H., Yagita H., Teng M.W., Smyth M.J. (2011). Anti-ErbB-2 mAb therapy requires type I and II interferons and synergizes with anti-PD-1 or anti-CD137 mAb therapy. Proc. Natl. Acad. Sci. USA.

[47] Rha S.Y., Lee C., Kim H.S., Kang B., Jung M., Kwon W.S., Bae W.K., Koo D.-H., Shin S.-J., Jeung H.-C. (2021). A multi-institutional phase Ib/II trial of first-line triplet regimen (Pembrolizumab, Trastuzumab, Chemotherapy) for HER2-positive advanced gastric and gastroesophageal junction cancer (PANTHERA Trial): Molecular profiling and clinical update. J. Clin. Oncol..

[48] Janjigian Y.Y., Kawazoe A., Yañez P., Li N., Lonardi S., Kolesnik O., Barajas O., Bai Y., Shen L., Tang Y. (2021). The KEYNOTE-811 trial of dual PD-1 and HER2 blockade in HER2-positive gastric cancer. Nature.

[49] Tsukita S., Furuse M. (2000). The Structure and Function of Claudins, Cell Adhesion Molecules at Tight Junctions. Ann. N. Y. Acad. Sci..

[50] Krause G., Winkler L., Mueller S.L., Haseloff R.F., Piontek J., Blasig I.E. (2008). Structure and function of claudins. Biochim. Biophys. Acta (BBA)—Biomembr..

[51] Bhat A.A., Syed N., Therachiyil L., Nisar S., Hashem S., Macha M.A., Yadav S.K., Krishnankutty R., Muralitharan S., Al-Naemi H. (2020). Claudin-1, A Double-Edged Sword in Cancer. Int. J. Mol. Sci..

[52] Tabariès S., Siegel P.M. (2016). The role of claudins in cancer metastasis. Oncogene.

[53] Pellino A., Brignola S., Riello E., Niero M., Murgioni S., Guido M., Nappo F., Businello G., Sbaraglia M., Bergamo F. (2021). Association of CLDN18 Protein Expression with Clinicopathological Features and Prognosis in Advanced Gastric and Gastroesophageal Junction Adenocarcinomas. J. Pers. Med..

[54] Kubota Y., Kawazoe A., Mishima S., Nakamura Y., Kotani D., Kuboki Y., Bando H., Kojima T., Doi T., Yoshino T. (2023). Comprehensive clinical and molecular characterization of claudin 18.2 expression in advanced gastric or gastroesophageal junction cancer. ESMO Open.

[55] Sahin U., Koslowski M., Dhaene K., Usener D., Brandenburg G., Seitz G., Huber C., Turecil O. (2008). Claudin-18 Splice Variant 2 Is a Pan-Cancer Target Suitable for Therapeutic Antibody Development. Clin. Cancer Res..

[56] Sahin U., Türeci Manikhas G., Lordick F., Rusyn A., Vynnychenko I., Dudov A., Bazin I., Bondarenko I., Melichar B., Dhaene K. (2021). FAST: A randomised phase II study of zolbetuximab (IMAB362) plus EOX versus EOX alone for first-line treatment of advanced CLDN18.2-positive gastric and gastro-oesophageal adenocarcinoma. Ann. Oncol..

[57] Türeci O., Sahin U., Schulze-Bergkamen H., Zvirbule Z., Lordick F., Koeberle D., Thuss-Patience P., Ettrich T., Arnold D., Bassermann F. (2019). A multicentre, phase IIa study of zolbetuximab as a single agent in patients with recurrent or refractory advanced adenocarcinoma of the stomach or lower oesophagus: The MONO study. Ann. Oncol..

[58] Rohde C., Yamaguchi R., Mukhina S., Sahin U., Itoh K., Türeci Ö. (2019). Comparison of Claudin 18.2 expression in primary tumors and lymph node metastases in Japanese patients with gastric adenocarcinoma. Jpn. J. Clin. Oncol..

[59] Moran D., Maurus D., Rohde C., Arozullah A. (2018). Prevalence of CLDN18.2, HER2 and PD-L1 in gastric cancer samples. Ann. Oncol..

[60] Sahin U., Schuler M., Richly H., Bauer S., Krilova A., Dechow T., Jerling M., Utsch M., Rohde C., Dhaene K. (2018). A phase I dose-escalation study of IMAB362 (Zolbetuximab) in patients with advanced gastric and gastro-oesophageal junction cancer. Eur. J. Cancer.

[61] Türeci Ӧzlem Mitnacht-Kraus R., Wöll S., Yamada T., Sahin U. (2018). Characterization of zolbetuximab in pancreatic cancer models. Oncoimmunology.

[62] Shitara K., Lordick F., Bang Y.J., Enzinger P., Ilson D., Shah M.A., van Cutsem E., Xu R.H., Aprile G., Xu J. (2023). Zolbetuximab plus mFOLFOX6 in patients with CLDN18.2-positive, HER2-negative, untreated, locally advanced unresectable or metastatic gastric or gastro-oesophageal junction adenocarcinoma (SPOTLIGHT): A multicentre, randomised, double-blind, phase 3 trial. Lancet.

[63] Xu R., Shitara K., Ajani J.A., Bang Y.-J., Enzinger P.C., Ilson D.H., Lordick F., van Cutsem E., Gallego Plazas J., Huang J. (2023). Zolbetuximab + CAPOX in 1L claudin-18.2+ (CLDN18.2+)/HER2− locally advanced (LA) or metastatic gastric or gastroesophageal junction (mG/GEJ) adenocarcinoma: Primary phase 3 results from GLOW. J. Clin. Oncol..

[64] Shah M.A., Kennedy E.B., Alarcon-Rozas A.E., Alcindor T., Bartley A.N., Malowany A.B., Bhadkamkar N.A., Deighton D.C., Janjigian Y., Karippot A. (2023). Immunotherapy and Targeted Therapy for Advanced Gastroesophageal Cancer: ASCO Guideline. J. Clin. Oncol..

